# Designing and psychometric of reproductive health related behaviors assessment tool in Iranian males: an exploratory mixed method study protocol

**DOI:** 10.1186/s12978-020-00966-z

**Published:** 2020-08-03

**Authors:** Mehrnaz Geranmayeh, Armin Zareiyan, Zahra Behboodi Moghadam, Mojgan Mirghafourvand, Fovziye Sanaati

**Affiliations:** 1 Department of Midwifery, Faculty of Nursing and Midwifery, Tehran University of Medical Sciences, Tehran, Iran; 2 Public Health Department, Nursing Faculty, AjA University of Medical Sciences, Tehran, Iran; 3 Reproductive Health Department of Reproductive Health Midwifery, School of Nursing & Midwifery Tehran University of Medical Sciences, Tehran, Iran; 4 Midwifery Department, Social determinants of Health Research Center, Tabriz University of Medical Sciences, Tabriz, Iran; 5 Department of Midwifery, Faculty of Nursing and Midwifery, Tehran University of Medical Sciences, Tehran, Iran

**Keywords:** Reproductive health, Mixed method, Qualitative method, Instrument, Tool, Men, Behavior

## Abstract

**Background:**

Male reproductive health is a relatively new concept, and most men are neglected in reproductive health discussions. Therefore, it appears that there is insufficient information about the male reproductive health. This study aims to design a psychometric instrument for assessing the male reproductive health-related behavior.

**Methods/design:**

This is a sequential exploratory mixed-method study with a classical instrument development design. It will be conducted in two qualitative and quantitative phases on the studied units including the men living in Tehran. In the first phase, a qualitative study of a contractual content analysis approach will be conducted in order to perceive the concept of male reproductive health-related behavior, determine the dimensions of the questionnaire, and explore the items. In the second phase, a quantitative study will be carried out to evaluate the psychometric properties as well as (form, content, and construct) validity and reliability of the instrument designed in the first phase. Finally, the instrument will be scored and interpreted.

**Discussion:**

Discovering men’s perception of concept of reproductive health-related behavior can help design a valid and reliable questionnaire which can be used in studies evaluating the male reproductive health-related behavior.

**Ethical code:**

IR.TUMS.FNM.REC.1397.157.

## Plain English summary

As defined by the World Health Organization (WHO) and the United Nations Fund for Population Activities (UNFPA), reproductive health refers to “a state of complete physical, mental, and social well-being and not merely the absence of disease or infirmity in all matters pertaining to the reproductive system and to its functions and processes.” Unlike women, reproductive health in men is a relatively new concept, and most men are ignored in reproductive health discussions. Typically, the reproductive health programs and services focus mostly on women and adolescents rather than adult men. Therefore, there is apparently insufficient information about men’s reproductive health. The levels of self-care and attention paid to reproductive health and awareness of the factors affecting reproductive health are lower in men than in women. Improving reproductive health in men not only improves their health but also has obvious effects on family health, including women’s and children’s health. In addition, it can improve mental health and reduce alcohol consumption and violence in men. It is necessary to identify the factors affecting men’s reproductive health, including behavioral factors, in order to improve their reproductive health because improving men’s behavior through health programs helps improve their reproductive health. This sequential exploratory mixed methods study of a classical instrument development design will be conducted in two qualitative and quantitative phases on the studied units, including men living in Tehran. The first phase is a qualitative study with a contractual content analysis approach and will be conducted to perceive the concept of reproductive health-related behavior in men, determine the dimensions of the questionnaire, and explore the items. The validity and reliability of the developed instrument will be analyzed in the second phase, i.e. the quantitative phase.

## Background

Health promotion is a process that enables people to control and enhance their health and well-being [1]. The concept of health puts more emphasis now on the behavior related to health and its protection so that the health of individuals, families, and society should be provided, maintained, and promoted through this behavior [2]. Health improvement focuses mostly on disease prevention and creating and adopting self-care skills and abilities [3].

As defined by the WHO and the UNFPA, reproductive health refers to “a state of complete physical, mental, and social well-being and not merely the absence of disease or infirmity in all matters pertaining to the reproductive system and to its functions and processes” [4]. Reproductive health is an important part of men’s overall health. Unlike women among whom reproductive health is well-known, it is a relatively new concept among men, most of whom are ignored in the discussions about reproductive health. Typically, the reproductive health programs and services focus mostly on women and adolescents rather than adult men. Therefore, there is insufficient information about men’s reproductive health. The levels of self-care and attention paid to reproductive health and awareness of the factors affecting reproductive health are lower in men than in women [5, 6]. Improving reproductive health in men not only improves their health but also has obvious effects on family health, including women’s and children’s health. In addition, it can improve mental health and reduce alcohol consumption and violence in men [7].

Various factors affecting men’s reproductive health should be addressed to improve and enhance their reproductive health. According to the literature, many factors can affect reproductive health in men, including sociodemographic characteristics (such as age, education, and socioeconomic status), lifestyle factors (such as obesity and overweight, alcohol consumption, smoking, physical activity, and diet), and biomedical risk factors (such as heart disease, diabetes, and drug treatments) [8–11] and environmental and behavioral factors [12–14]. A majority of unhealthy behavior can directly affect reproductive health and increase fertility-related disorders among men by lowering sperm and semen quality [15, 16]. Alcohol consumption, smoking, poor nutrition, and overweight/obesity are instances of the unhealthiest behavior that threaten men’s fertility status and children’s health. These risk factors not only affect the fertility of men but also put their disability-adjusted life year (DALYs) at risk and increase the rates of morbidity and mortality [8, 17, 18]. Instances of unhealthy behavior such as overweight/obesity and the resultant problems increase the incidence of prostate cancer in men [19]. In addition to reproductive health, some cases of unhealthy behavior that lead to overweight and obesity can negatively affect men’s health and sexual function. For example, studies have shown that a high-fat, high-calorie diet as well as overweight and obesity are associated with erectile dysfunction [11, 20, 21]. At the same time, unhealthy behavior and lack of *exploratory* health-related behavior are more prevalent in men than in women [22, 23]; hence, unhealthy behavior can have more destructive effects on reproductive health in men than in women [14].

In recent years, there has been a growing trend in men’s reproductive health-related problems. In many European countries, semen quality of young men was at least 20% below the WHO reference level, affecting the fertility rate ([24–26]. Moreover, instances of healthy behavior such as exercise, healthy eating habits and normal weight, and avoidance of unhealthy behavior help increase fertility in couples [27, 28]. Therefore, government health programs should emphasize on the promotion of reproductive health among men as a large part of the population and a basic element in family health. Men should be empowered to control and increase their health [29–31]. Given the limited number of studies on men’s health, health-related behavior has been investigated in some countries in recent decades with the purpose of improving men’s health [32–34]. For instance, Zhang et al. (2015) examined the reproductive health-related knowledge, attitudes, and performance of Chinese men [34]; Sawyer et al. (2004) evaluated the reproductive health-related knowledge, attitudes, and performance of men with cystic fibrosis in the Cystic Fibrosis Clinic of Children’s Hospital in Boston, Massachusetts (USA) [35]. A telephone survey in Australia (2005) assessed men’s reproductive health and its associated concerns [36, 37]. Since the instances of reproductive health-related behavior are influenced by social norms, culture, mass media, national health policies, promotional performance, and physical and social environments, specific criteria and developing instruments for assessing reproductive health-related behavior seem essential for improving and promoting male reproductive health. Such instruments can help identify this behavior in men and improve their reproductive health through behavioral modification achieved by training or reinforcing plans. Given the fact that there is little information and few specific instruments in the field of men’s reproductive health-related behavior, the researcher intends to conduct a combined multiphase sequential exploratory study to develop an instrument for assessing the reproductive men’s health-related behavior and to evaluate its psychometric properties in the hope of taking a step to improve reproductive health of this large population.

### Objectives

This study aims to develop an instrument for assessing men’s reproductive health-related behavior in men and to evaluate its psychometric properties.

The specific research objectives are as follows:

Specific objectives of the qualitative phase (Phase 1):

1. Explaining men’s perception of reproductive health-related behavior
2. Developing items of the men’s reproductive health-related behavior instrument

Secondary objectives of the quantitative phase (Phase 2):

1. Determining the formal validity of the men’s reproductive health-related behaviors instrument
2. Determining the content validity of the men’s reproductive health-related behaviors instrument
3. Determining the construct validity of the men’s reproductive health-related behaviors instrument
4. Determining the internal consistency of the men’s reproductive health-related behaviors instrument
5. Determining the (relative and absolute) stability of the men’s reproductive health-related behaviors instrument
6. Determining the Weight status of the men’s reproductive health-related behaviors instrument
7. Determining the scoring status of the men’s reproductive health-related behaviors instrument

## Materials and methods

### Study design

**Fig. 1:**
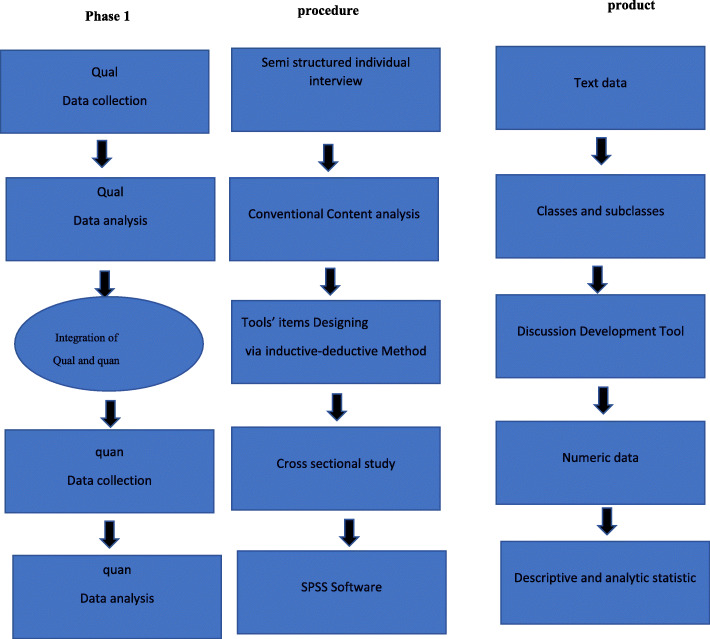
Study visual diagram

This is a sequential exploratory mixed-method study with a classical instrument development design. The mixed-method studies are often based on the pragmatism philosophical approach. Based on this approach, the mixed use of qualitative and quantitative methods leads to a better perception of the studied phenomenon. A sequential exploratory design is a biphasic mixed study in which the researcher qualitatively explores the intended subject before constructing the quantitative study [38]). This study will consist of two phases and a chronological sequence for performing qualitative and quantitative phases sequentially so that, for in-depth and comprehensive recognition of the phenomenon, the qualitative data will be collected first, and the quantitative data will then be collected and analyzed. Since this study ultimately aims to develop an instrument, the qualitative phase takes precedence over the quantitative phase, and the qualitative part weighs more than the quantitative part (Fig. 1).

#### Phase 1: qualitative part of the study

This phase consists of a qualitative study on the contractual content analysis type. First, the researcher will use an inductive approach to explain men’s perception of reproductive health-related behavior and to explore the instrument’s items in proportion to the Iranian cultural and social contexts. For this purpose, the men meeting the inclusion criteria will undergo semi-structured, in-depth, and individual interviews; sampling and interviewing at this phase will continue until the data saturation level, which is reached when no new data are added. The resultant data of qualitative interviews will then be analyzed through the contractual content analysis method in eight stages based on the method proposed by Zhang and Wildemuth (2009) [39]. At the end of this phase when the concept of men’s reproductive health-related behavior is explained, the researcher will inductively develop the questionnaire items and will then comparatively complete the instrument items through extensive reviewing of studies in this field. Therefore, a preliminary instrument will be developed to assess men’s reproductive health-related behavior.

### Sampling and selection of participants

Objective-based sampling with maximum diversity will be used for sampling in the first phase of the study. The researcher goes to the health centers affiliated with Tehran University of Medical Sciences to find the primary participants. The next participants will be selected by considering the results of continuous comparative analysis and asking the samples questions, taking into account the maximum diversity and different characteristics (in terms of age, education, etc.).

### Participants and inclusion criteria

In the qualitative phase, the participants will include literate men living in Tehran with no infertility problems.

### Data collection

After the approval from the Ethics Committee of Tehran University of Medical Sciences and the required permissions are granted, an in-depth semi-structured personal interview will be conducted using the interview guide questions regarding men’s perception of reproductive health-related behavior. The interview will be conducted at the time and place convenient for the participants after their consent is obtained. The researcher will explain the research importance and objectives in an understandable language to the participants, and will record the interview in the written or oral form after ensuring the confidentiality of their conversations and obtaining their permission. Personal information of the participants such as age, education, occupation, and work experience will be collected through a questionnaire. The participants will then be asked to express their concerns about their reproductive health as well as their experiences of reproductive health-related behavior. Since the interviews will be conducted in a semi-structured manner, the interview guide questions will be based on the interview process.

Therefore, the next questions will be asked based on the initial answers of the participants and the interview guide. The number of sessions will vary depending on the research responses. The interviews will be recorded and then transcribed word by word.

### Determining the scientific validity and reliability of data

To ensure the acceptability of the data collected in the qualitative phase, the transcripts of interviews will be reviewed by the research participants (member check), revised by the supervisors (external check and peer debriefing), and continuously evaluated by the researcher (prolonged engagement), and sampling will be performed with maximum variety.

In order to ensure the research reliability, the results will be provided for an external supervisor to conduct the research audit.

The transcripts of interviews and codes extracted by the participants, the research team members, and the external supervisor will be reviewed to verify the research findings.

In addition, the researcher will try to precisely record the research path and the decisions made in this regard, enabling other researchers to follow the research path and work steps. Moreover, in order to transfer more data in this study, the samples will be selected with maximum diversity in terms of age, education, etc. The research accuracy will be confirmed using the researchers’ credibility, i.e. reviewing and revising the interviews, codes and categories extracted by the research team members, long-term interaction with the participants, and maintaining continuous communication with the corresponding author’s notes.

### Data analysis

In this study, the content of qualitative data will be analyzed through the content analysis method proposed by Zhang and Wildemuth (2009) in eight steps as follows.

In the first step, the data will be prepared for the qualitative content analysis. The recorded interviews will then be transcribed, and the participants’ nonverbal messages such as the tone of voice, silence, and crying noted during the interview will be added to the transcripts because some concepts and patterns are hidden inside the data which to be extracted.

The second step is to decide on the analysis unit. The analytical or semantic unit is in fact the basic piece of the text that is classified and coded during the content analysis. Therefore, one of the most important and fundamental decisions of content analysis is to determine three units. Individual themes including words, sentences, and paragraphs within the text are usually used in the qualitative content analysis. Therefore, the text should be searched for sentences or expressions from which the themes are derived.

The third step pertains to classification and coding, which will be inductively extracted from the data by continuous comparison of the categories. The codes are placed in the subcategories according to their similarity, then the categories are formed based on the relationship of subcategories to each other, and the categories are organized in such a way that they have internal consistency and external inconsistency.

In the fourth step, the codes are tested in a sample of the text so that a sample of the text will be coded by the researcher and that the data coding stability will be controlled by two research team members.

In the fifth step, after the researcher and two members of the research team agree on coding stability, a repeatable process is achieved and the coding process is extended to the whole text.

The sixth step is to achieve the coding stability. In order to control any human errors such as fatigue or misunderstanding, and people’s perception of categories and coding rules, which may change over time and lead to instability, coding stability (initial codes, placing them in subcategories, and formation of categories) is double-checked by two members of the research team and the experienced people in the field of qualitative research, as a continuous process during content analysis. This is because new themes and concepts might be added to the text.

The seventh step pertains to conclusions obtained from the categorized and coded data. The characteristics and dimensions of the categories are discovered, and the relationships of categories are identified. The hidden patterns are then revealed, and the categories are tested in a wide range of data so that the categories and subcategories will be compared in the related text. The input and output of the data are extracted and examined to verify whether the main categories or the themes are formed from the essence of the data. Finally, in the eighth stage, the formed categories will be reported.

#### Phase 2: the quantitative part of the study

At this phase, the psychometric properties of the developed instrument will be examined through the methodological study.

### Research samples

In the quantitative phase, the participants will be different from those of the qualitative phase. The quantitative phase requires a large sample size of participants, and the researcher will perform statistical tests after collecting information from them by using the instrument developed in the qualitative phase to provide an acceptable result.

### Inclusion criteria

Inclusion criteria in this phase of the study will include literate men living in Tehran who are willing to participate in the research and fill out the questionnaire.

### Exclusion criteria

Men with infertility problems will be excluded from the study.

### Data collection

In the quantitative phase, the data will be collected through a questionnaire that will be developed after the qualitative study. The questionnaires will be completed through self-reporting.

### Sample size

Based on the items of the instrument developed after the initial qualitative study, the sample size of the quantitative study will be determined as follows:

- For the formal and content validity of item, at least 10 participants will be enrolled in the study.
- For the initial reliability before factor analysis through the internal consistency method with Cronbach’s alpha coefficient, 20 participants will be enrolled in the study.
- For the construct validity, 3–10 participants will be enrolled in the study for each item.
- The test-retest reliability will be checked with 20 participants and the final Cronbach’s alpha coefficient will be performed for the extracted dimensions with the same number of participants for the construct validity [40].

### Data analysis

In a quantitative phase, the obtained data will be analyzed in SPSS 21 using descriptive and analytical indices such as Cronbach’s alpha, the Pearson correlation test, intra-cluster correlation, and exploratory factor analysis.

### Determining the scientific validity and reliability of the data

#### First stage: determining the questionnaire validity

##### Formal validity determination

The formal validity of the instrument will be determined through both qualitative and quantitative methods [40, 41].

##### Qualitative determination of formal validity

In this method, 10 participants will be asked to fill out the questionnaire to obtain a formal validity and comment about the difficulty (in understanding phrases and words), the irrelevance (appropriateness of the phrases and a favorable relationship between the phrases and the main purpose and dimensions of the questionnaire), and ambiguity (possible misinterpretation of phrases or misunderstanding of words). The comments will then be used to modify the questionnaire [40, 41].

##### Quantitative determination of formal validity

In this section, the item impact index will be employed to reduce and eliminate inappropriate phrases and determine the importance of each phrase. The item impact index is utilized to measure the importance frequency of items remained and modified from the initial pool of items. This will discard the items that are considered relatively insignificant or irrelevant by the participants. To this end, 10 participants will score the importance of the items based on the 5-point Likert scale as completely important (5), somewhat important (4), moderately important (3), slightly important (2), and not important at all (1). The researcher then will calculate the impact score of each item separately using the following formula:

$$ \mathrm{Importance}\times \mathrm{Impact}\ \mathrm{Score}=\mathrm{Frequency}\ \left(\%\right) $$

Frequency (in percent) refers to the number of individuals who scored 4 and 5 on each item, and importance refers to the mean score of importance based on the Likert spectrum. If the impact score is greater than 1.5, the phrase is relevant and will be maintained for further analysis [40].

##### Content validity determination

The content validity of the instrument will be determined through both qualitative and quantitative methods [40, 41].

To determine the qualitative content validity, the researcher will ask a group of reproductive health researchers and professionals to give their expert opinions about the instrument in terms of the grammar, proper word placement, proper item placement, and appropriate scoring after carefully studying the instrument. The instrument will be modified according to them.

To determine the quantitative content validity, the two coefficients of content validity ratio (CVR) and content validity index (CVI) will be used based on kappa statistics. Moreover, the CVR and CVI will be calculated numerically by using the critical value table proposed by Zareian and kappa statistics, respectively.

The CVR assesses the need for items of the reproductive health-related behavior instrument from the perspective of the panel of experts. For this purpose, they will be asked to review each item based on the 3-point Likert spectrum (necessary, useful but unnecessary, and unnecessary). Then CVR will be calculated based on the following formula:

$$ \mathrm{CVR}=\left({\mathrm{n}}_{\mathrm{E}}=\mathrm{N}/2\right)\div \mathrm{N}/2 $$

Where N is the total number of experts and n_E_ is the number of experts who choose the necessary option. CVRs calculated from this formula and obtained from the CVR table will be compared and the decision will be made. The content validity of items will be confirmed if the calculated CVR is greater than the table CVR.

Criteria for selecting an appropriate CVI for instrument-developers include focusing on estimating agreement compared with consistency, ease of calculation, understandability and ease of content transfer to others, providing information about both the item and the whole instrument, and adjust for chance agreement elimination. Except the last criterion, i.e. adjustment for chance agreement elimination, the CVI has other criteria. Therefore, Polit, Beck, and Owen (2017) [42] proposed a new method of using the corrected kappa statistics with the K* sign, since it provides an indicator of the chance agreement between the evaluators as relevancy of the item. CVI is calculated first through computing the probability of the chance agreement using the following formula, which is used for binominal random variables.

$$ {\mathrm{P}}_{\mathrm{c}}=\left[\mathrm{N}!\div \mathrm{A}!\left(\mathrm{N}-\mathrm{A}\right)!\right]\times {0.5}^{\mathrm{N}} $$

Where N is the number of evaluators and A is the number of agreements related to the item relevance [40, 43].

CVI will then be examined in the 4-point Likert spectrum for each item by 10 professionals (e.g. 1: irrelevant; 2: somewhat relevant; 3: relevant; and 4: quite relevant). For this purpose, the CVI score will be calculated by combining the agreement scores for each item ranked 3rd and 4th (highest score) by the total number of specialists.

Finally, K* will be calculated using the agreement ratio for the relevance of each item (I-CVI) and the probability of the chance agreement as follows. According to Lynn and Polit, the minimum number of evaluators should be three for calculating kappa through this method; the number of evaluators will be 10 in the present study. Kappa values of 0.59–0.40, 0.74–0.60, and > 0.74 will be evaluated poor, good, and excellent, respectively. In this study, only items with kappa of at least 0.74 will be accepted.

$$ \mathrm{K}\ast =\left(\mathrm{I}-\mathrm{CVI}-{\mathrm{P}}_{\mathrm{c}}\right)\div \left(1-{\mathrm{P}}_{\mathrm{c}}\right) $$

In addition, S-CVI (mean of I-CVIs for the whole instrument) of > 0.90 is the optimal criterion for content validity [40, 43].

##### Construct validity determination

In the present study, the construct validity will be determined through the exploratory factor analysis method [38, 40, 41, 44].

#### Second stage: determining the questionnaire reliability

Reliability of the instrument will be determined in two stages.

##### Initial reliability measurement

This stage intends to examine the whole instrument reliability and to identify the items that are likely to affect the reliability. This will identify the possible problems and will facilitate factor analysis through eliminating or correcting the items that may reduce reliability. To this end, Cronbach’s alpha coefficient is used. At this stage, the Cronbach’s alpha coefficient will be calculated by sampling at least 20 eligible individuals 40, 41].

##### Final reliability measurement

Two methods of determining internal consistency and stability will be employed to determine the instrument reliability.

Internal consistency: At this stage, the Cronbach’s alpha coefficient will be calculated by sampling at least 20 of the eligible conditions. Conventionally, a minimum alpha of 0.6 for exploratory studies, > 0.7 for confirmatory studies, and > 0.8 indicates good convergence. In general, to have a good internal consistency, the Cronbach’s alpha should be between 0.70 and 0.80 [40, 41].

Stability: The test-retest method will be utilized to evaluate the relative stability. For this purpose, the researcher will present the instrument to at least 20 people in 2 steps and will compare the scores obtained in these 2 steps. The time interval between the two tests should be such that not only are the instrument phrases forgotten, but also no change occurs in the phenomenon being measured. The interval between two steps usually lasts for 2 weeks, and a fixed number of people should conduct the test in two steps. The Pearson correlation coefficient and the intra-class correlation coefficient (ICC) will be employed to calculate the reliability coefficient. The ICC is an estimate of the agreement between the scores of two or more evaluators who evaluate a scale. Ranging between zero and 1, the ICC is good if it is 0.61–0.81 and excellent if it is > 0.81 [40, 45].

To measure the absolute stability, the standard error measurement (SEM) will be calculated. SEM shows whether the difference in measurement between the two tests is real or due to a measurement error. The researcher can achieve the confidence interval to estimate the range of scores in which the individual’s actual score is placed by calculating the SEM. The lower the SEM, the higher the reliability.

$$ \mathrm{SEM}=\mathrm{SD}\times 1-\mathrm{ICC} $$

##### Weight

In this study, the findings of factor analysis will be used considering the ratio of variances in each factor as well as the amount of factor load of each item to weight the items based on current mathematical formulas [46].

##### Scoring

The Likert scale and the linear conversion scoring method will be used in this study for scoring and interpretation of the instrument [40, 47].

## Discussion

Male reproductive health, affected by many behavioral and environmental factors, is often overlooked. Nearly 37% of men smoke during fertility age, a lifestyle habit which has a significantly negative effect on sperm health [48]. In addition, other lifestyle habits such as long stance and inactivity can reduce male reproductive health [49]. Identifying these factors in men and reducing their destructive effects through educational and supportive programs can help improve their health.

This study has several advantages. It can fill some of the research gaps in male reproductive health; therefore, it is expected to have important clinical implications. Developing a questionnaire for men’s reproductive health-related behavior can pave the way for further plans and measures. This study is also based on a combination of methods; therefore, it can support the integration of different and even contradictory approaches and methods. Collecting qualitative and quantitative data will help better perceive men’s experiences with cases of reproductive health-related behavior. There are only a few studies on male reproductive health and related behavior, none of which have developed a questionnaire. This protocol has also certain weaknesses, including sampling in only one city of Iran. To mitigate this weakness, sampling will be performed in both study phases with maximum diversity. Since infertile men are more likely to experience misbehavior, healthy men with no infertility problems will be included in the study.

## Data Availability

Not applicable.

## Abbreviations

WHO: World Health Organization
United UNFPA: Nations Fund for Population Activities
DALYs: disability-adjusted life year
CVI: Content Validity Index
CVR: Content Validity Ratio
SEM: Standard error measurement
